# Digital Health Innovation: A Toolkit to Navigate From Concept to Clinical Testing

**DOI:** 10.2196/cardio.7586

**Published:** 2018-01-18

**Authors:** Francoise Adeline Marvel, Jane Wang, Seth Shay Martin

**Affiliations:** ^1^ Ciccarone Center for the Prevention of Heart Disease Division of Cardiology, Department of Medicine Johns Hopkins University School of Medicine Baltimore, MD United States; ^2^ Johns Hopkins University School of Medicine Baltimore, MD United States

**Keywords:** digital health innovation models, mHealth, innovation framework, development of smartphone applications, wearable technology, healthcare transformation

## Abstract

Digital health technologies such as smartphone apps, Web-based platforms, and wearable devices are rapidly emerging as promising interventions for acute and chronic disease management, particularly in the field of cardiovascular medicine. However, there is limited guidance on how to effectively develop and rigorously test digital health interventions (DHIs). Through our experience with innovating Corrie, a smartphone-based app paired with a smartwatch and blood pressure monitor for myocardial infarction recovery in the acute setting, we aim to provide a toolkit for navigating the digital health technology development and clinical testing processes. The toolkit consists of 6 steps: step one emphasizes concept generation by defining a specific clinical problem and the existing solutions aimed to address it; step two aims to recruit a multidisciplinary team within an academic institution; step three leverages technology accelerators and industry partnerships; step four develops the digital health technology with continuous feedback from patient and family end-users; step five solicits feedback from a diverse array of stakeholders; and step six performs a clinical study at a single site that, if successful, rapidly scales to multiple sites. DHI development is often a complex and vastly uncharted territory. By exploring the steps we took from concept to clinical testing with the first cardiology CareKit app, we hope to provide useful insights to teams that are starting out on their path to digital health innovation. We emphasize the central importance of embracing transdisciplinary work to move from silos to synergy.

## Introduction

Cardiovascular disease management is being redefined by the unprecedented innovation in digital health technologies for health care delivery, patient empowerment, and engagement. The opportunities for developing, testing, and disseminating digital health interventions (DHIs) continue to grow exponentially as industry forecasters predict that global smartphone ownership will reach 90% by 2020 [1]. DHIs are defined as any form of software or hardware app used to improve the quality, access, efficacy, or efficiency of health care delivery [2]. DHI modalities span a wide range, from mobile apps to telemedicine, email, text messaging services, and wearable devices [3], and are being used in a variety of contexts, such as smoking cessation [4], behavioral modification [5], and weight loss [6]. Cardiology-focused digital health technologies, as in other fields, remain in beta testing [7]. Such technologies have provided potential glimpses into the future, and encouraging early results support the feasibility of large-scale, smartphone-based lifestyle measurement [8]. Provocative early studies further suggest that text messaging can positively impact physical activity [9], cardiac risk factors [10], and medication adherence [11].

Momentum is building for DHIs to reinvent cardiovascular care delivery. A Johns Hopkins-based smartphone app, *Corrie Health*, is an example of such a DHI that is transforming the hospital discharge process by delivering the key components of discharge instructions early during hospitalization via an engaging patient-centered app and smartwatch, rather than in static paper form during the final minutes of hospitalization. This approach represents a complete reengineering of the discharge process to empower patients in skill-building, monitoring, access to care, and self-management to promote high-value care as patients make the risky transition from hospital to home.

Both academia and industry are driving forces in this emerging field, but the limitations in their digital health products suggest a need for even greater collaboration. For example, apps originating from industry often lack behavioral theoretical basis and clinical foundation [12], and academia faces challenges with keeping updated with technological advancements and end-user experience [13]. The creation of DHIs requires intensive collaborative efforts. Just as technology development leaders may be sequestered in silos of computing and engineering, health care experts are often isolated in centers of biomedical research and academic hospital settings.

**Figure 1:**
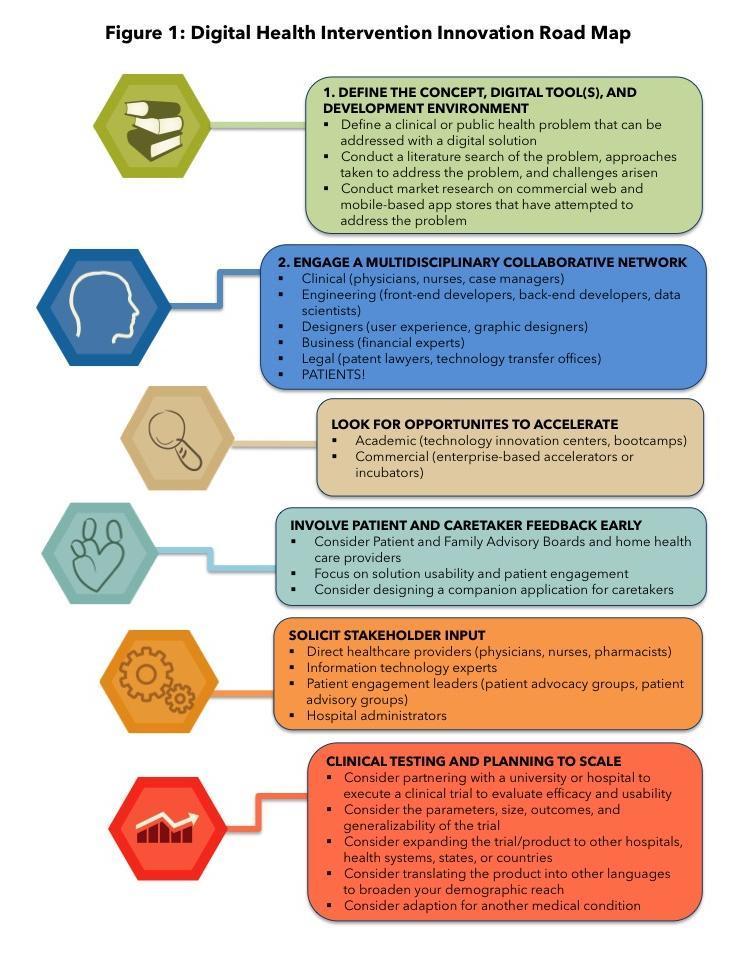
Digital health intervention innovation roadmap.

To bridge the gap between technology development and health care, it is particularly valuable to have a roadmap for developing safe, effective, and evidence-based DHIs, and incorporating mobile technology into health care based on an interdisciplinary perspective. Here we aim to contribute a step-wise approach to smartphone-based DHIs with practical guidance for each step. By delineating the steps we took from concept to clinical testing (Figure 1), we seek to provide useful insights to teams that are interested in building a smartphone app and starting out on their path to digital health innovation.

## Innovation Toolkit (Steps 1-6)

### 1. Define the Problem, Concept, and Digital Tool(s)

Begin by defining a distinct clinical problem that may be addressed with a DHI. Conduct a literature search to investigate the current understanding of the issue, the methods that researchers have used to address the question, and the challenges that have arisen throughout their experiences. Simultaneously conduct market research on Web- and mobile-based app stores for solutions that have already been produced, taking note of their strengths, weaknesses, and receptivity in order to understand how innovative your concept is. For example, with *Corrie*, research was conducted on the Apple iTunes Store [14], Google [15], Skyscape [16], and Unbound Medicine [17] at the outset of the project using the terms ‘‘cardiology,’’ ‘‘self-management,’’ ‘‘medications,’’ “inpatient,” and ‘‘follow-up.’’ This search generated approximately 125 apps. Similar apps identified in the search were analyzed by their functional capabilities, target condition, content, layout, and design. We found no apps that combined medication management, education, follow-up coordination, and inpatient deployment for acute myocardial infarction recovery.

**Figure 2:**
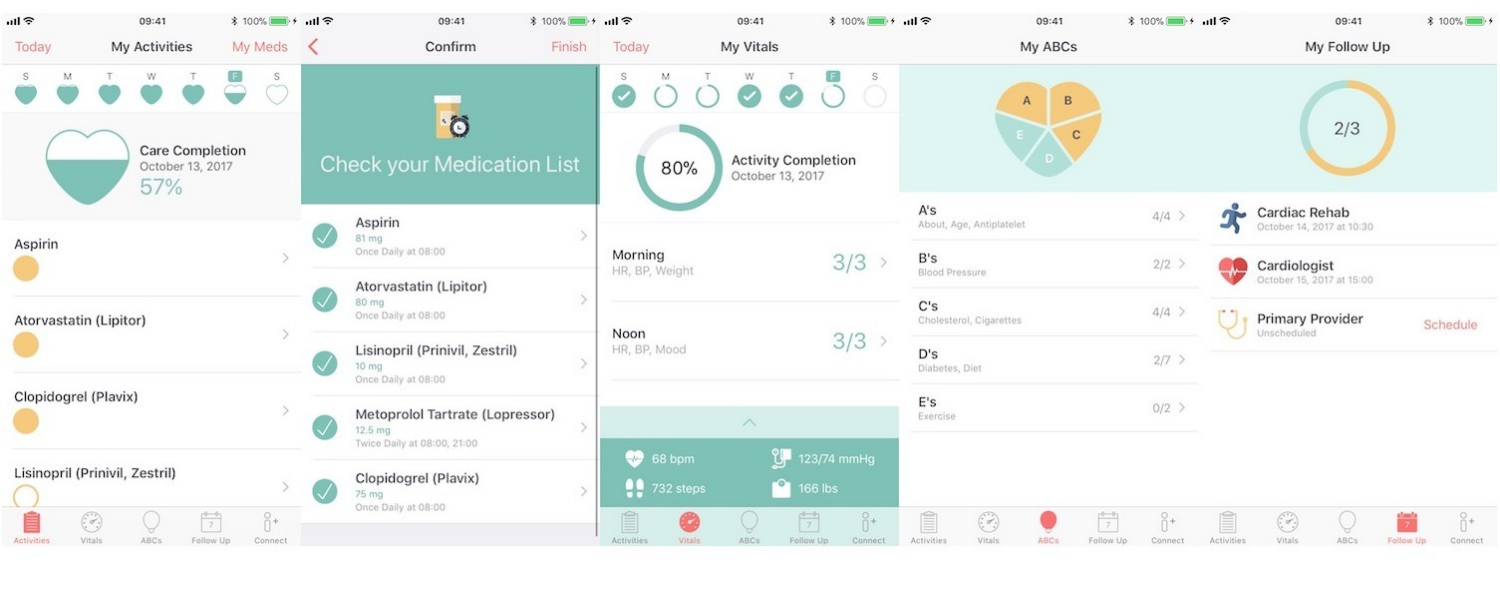
Corrie Health app screenshots.

To build a solution, there are 3 large categories of mobile tools to draw from: mobile apps, short message service (SMS) messaging, and wearables/sensors. Consider device and software integrations and how to link data monitoring with action. The development context may be selected from native app development in Apple, Android, Windows/Nokia, Blackberry, or cross-platform (eg, Ruby and Rails, Appcelerator, PhoneGap) that can be used across platforms as HTML-based apps. Although there are multiple platforms, iOS and Android account for 99.6% of all smartphone sales, according to the latest industry report [18]. In our experience, we started with a cross-platform build and later transitioned to native app development, which allows utilization of intrinsic smartphone features (eg, camera, contacts, calendar); this created a significant advantage for feature scope and an improved user interface (see Figure 2).

### 2. Build a Multidimensional Collaborative Team

Digital health calls for interdisciplinary teamwork, which is an aspect that renders it challenging but also uniquely rewarding. Members of a team may include physicians, nurses, pharmacists, case managers, and engineers, as well as experts in behavioral science, graphic design, informatics, human-computer interaction, business, and last but not least, patients.

We built our internal team primarily by networking within our university. To recruit the clinical team we aimed for cardiologists, internal medicine physicians, housestaff, nurses, medical students, premedical students, and pharmacists; all of whom possessed an interest in digital health, innovation, implementation research, and health care transformation. We sent recruitment emails via departmental electronic mailing lists and also gave numerous project presentations at digital health interest groups, academic teaching conferences, and individual faculty audiences. A growing number of academic centers, including Johns Hopkins (where *Corrie* was created), have opportunities for collaboration through Technology Innovation Labs [19,20], Biomedical Engineering Programs [21,22], and Healthcare Transformation Labs [23]. Our *Corrie* team opted to recruit expertise within the Johns Hopkins University system, which allowed for dynamic bidirectional collaboration in real time, rather than funding an outsourced mobile health (mHealth) app development team to create a product based on specifications.

Similar to the clinical team recruitment, we recruited technical expertise for software, frontend, and backend requirements of a project with recruitment emails, presentations at Technology Innovation interest groups, and individual presentations.

Of note, given the importance of data security and Health Insurance Portability and Accountability Act (HIPAA) regulations for patient health information, it is key to have a technical engineer skilled in data security, data management, and backend engineering to meet the requirements for data security at the university and institutional review board (IRB) levels. The health system’s HIPAA compliance office can be a resource as questions arise.

Business expertise may be required to build financial models of how the product might sustain itself before and after reaching the market. Early on, innovators at academic centers should also engage their Technology Transfer Office, which provides guidance with intellectual property protection (potential patents and/or copyrights), disclosure of inventions, and commercialization opportunities. Additional resources for team building can be found by participating in professional mHealth training networks [24] and health transformation fellowships [25].

### 3. Accelerate Development of Technology and Diffusion of Innovation

Once a multidisciplinary team is established, the next step is to begin building the solution as efficiently and effectively as possible due to the rapidly evolving nature of digital health. As we started to build a native iOS App, Apple Inc. (Cupertino, California) introduced an open-source framework for clinical data collection in 2015 called *ResearchKit* [26], and in 2016 the company released a patient-facing framework with modules for care plan visualization, symptom and objective measurement tracking, and care team engagement called *CareKit* [26]. We found that the goals of these initiatives were aligned with ours and that using these software frameworks accelerated our development. The *Corrie* team sought opportunities to accelerate development, and at early stages we participated in a clinical and technology development accelerator [27], a bootcamp in technology development [28], and a patient safety and quality improvement fellowship at Johns Hopkins [29].

Funding acquisition is another important aspect to fuel project momentum. To fund the time of software engineers, we applied for innovation grants (academic and state-based), business grants (ie, seed grants for health care innovation), and patient safety and quality improvement funding. Both academic and industry health transformation challenges (ie, “shark tanks”) were additional funding sources.

### 4. Involve Patients and Families Early and Often Throughout Development

Patients will only use new technologies if they find them relevant, engaging, and easy-to-use. Also important are the needs of those who are peripherally involved and affected, including partners, family members, and caretakers. In developing *Corrie*, we engaged acute myocardial infarction patients as partners early in the design process through the following pathways: (1) performing demonstrations for feedback at Patient and Family Advisory Boards [30]; (2) recruiting patients who were end-users of *Corrie* to serve on our own Patient Advisory Board; and (3) inviting patients to participate as part of our research team, which is a growing trend in digital health innovation [31]. To keep patients and families connected and invested in *Corrie’s* ongoing development, we created a monthly e-newsletter as well as *Corrie* Facebook, Instagram, and Twitter social media outlets.

### 5. Seek Feedback From a Diverse Array of Stakeholders

While patient and caretaker feedback is a priority, many other stakeholders can provide valuable input into a DHI. We sought a diverse range of feedback from ground level health care providers, informational technology experts, patient engagement leaders, and hospital administrators. Each group provides a unique perspective on critical components for the solution to work within the system. To successfully connect with stakeholders and facilitate a meaningful collaboration, it is extremely beneficial to have a well-designed introductory slidedeck consisting of 10 slides that explain the significance of the problem, how your solution addresses it, a summary of your solution, and the potential impact, which concludes with your “asks” for the stakeholder (eg, feedback, recruitment, funding). Maintaining updated slide sets, as well as having an introductory video and website, can help disseminate the project and facilitate connections.

### 6. Perform Clinical Testing

Each prior step is preparation for this final one, which is critical for adoption. Rigorous clinical testing remains the exception rather than the rule in digital health. However, there is now a deep need for high-quality evidence generation to start catching up with the pace of technology. Innovative trial designs, such as microrandomization (or n-of-1 randomization), may help to build effective interventions more quickly. However, we cannot skip fundamental steps like establishing that our app’s measurements are accurate or that our platform is usable. Therefore, our clinical evaluation of *Corrie* is beginning with a focus on validation, feasibility, and usability in the Johns Hopkins *Myocardial infarction COmbined-device Recovery Enhancement* (MiCORE) study [32]. Between the piloting stage of work and efficacy trials powered for clinical events, there is a wide, open space in which much can be done to understand impact (ie, proximal patient-centered outcomes such as knowledge, engagement, activation, and satisfaction).

We had the advantage of working within a health system and therefore a direct path to patient testing. For innovators working outside of a health system, consider partnering with a local university or hospital to get your solution in the hands of patients to validate feasibility, usability, and efficacy.

Take time to consider the parameters, size, outcomes, and generalizability of your trial. One key consideration is the demographic of your trial population. Elderly populations are less likely to use mobile technology, yet have a higher prevalence of chronic conditions [33]. For example, *Corrie’s* target age group is 40 to 85 years old, so we collaborated with Apple to optimize intuitive design and user-friendly interfaces that emphasized color contrast, larger buttons and text size, and also minimized hierarchy (ie, number of screens to navigate through for each feature).

Powered by automation, digital health technologies lend themselves to scaling, which offers a significant advantage for dissemination of solutions in this space. However, scaling a digital technology is not easy; it is likely a multistage process with a variety of options to consider. One can scale up, for example, by taking the solution to another hospital, state, or country. One can translate the solution into another language by adapting it for another culture, or adapt it for another health condition. In our experience, we have grappled with balancing the potential benefits of scaling versus the risk of diluting our efforts. Creating processes such as data security/storage and patient use agreements across all components of the app to use outside of the primary institution from which it was developed is important. It is also important to develop a sustainable business model to address the reimbursement and policy landscapes.

## Future Directions

Digital health technologies are poised to transform patient care. One significant barrier to the adoption and diffusion of such technologies is insufficient collaboration between key stakeholders in developing and testing the most promising DHIs. In this article, we described our firsthand experience with developing a DHI through a step-wise approach. In the future, to make this pathway more seamless, we recommend: (1) early multidisciplinary accelerators compromised of a variety of stakeholders, such as patients, physicians, nurses, technologists, informaticians, designers, business leaders, administrators, and cross-industry leaders; (2) establishment of institutional navigators who can provide a pathway through institutional roadblocks and operational factors; (3) encouraging mentorship and championship from faculty-level and administration; (4) devotion of administrative/business/finance leadership to create sustainable business models to address the reimbursement and policy landscapes; (5) creation of expedited IRB pathways for low-risk DHIs; and (6) the design of a systematic processes to access patient evaluations of new technologies and consumer-centered design.

## Abbreviations

DHI: digital health intervention
HIPAA: Health Insurance Portability and Accountability Act
IRB: institutional review board
mHealth: mobile health
MiCORE: Myocardial infarction COmbined-device Recovery Enhancement
SMS: short message service
